# Evaluation of FRAX in patients with periprosthetic fractures following primary total hip and knee arthroplasty

**DOI:** 10.1038/s41598-023-34230-8

**Published:** 2023-05-02

**Authors:** Lukas A. Holzer, Lisa Borotschnig, Gerold Holzer

**Affiliations:** 1 Department of Orthopaedic Surgery, AUVA Trauma Center Klagenfurt, Waidmannsdorferstraße 35, 9020 Klagenfurt am Wörthersee, Austria; 2 Department of Orthopaedics and Trauma, Medical University of Graz, Graz, Austria; 3 Department of Orthopaedics, Fiona Stanley Fremantle Hospitals Group, Perth, Australia; 4 Perth Orthopaedic and Sports Medicine Centre, Perth, Australia; 5 Department of Orthopedics and Traumatology, Medical University of Vienna, Vienna, Austria

**Keywords:** Musculoskeletal system, Medical research

## Abstract

The fracture risk assessment tool (FRAX) is a tool which calculates an individual 10-year fracture risk based on epidemiological data in patients with a risk of osteporosis. The aim of this study was to evaluate the value of FRAX to estimate the risk of postoperative periprosthetic fractures (PPF) in patients following with total hip and knee arthroplasty. 167 patients (137 periprosthetic fractures in total hip arthroplasty and 30 periprosthetic fractures in total knee arthroplasty) were included in this study. Patients’ data was retrieved retrospectively. In each patient the 10-year probability of a major osteoporotic fracture (MOF) and an osteoporotic hip fracture (HF) was calculated using FRAX. According to the NOGG guideline 57% of total hip arthroplasty (THA) patients and 43.3% of total knee arthroplasty (TKA) patients were in need of osteoporosis treatment, whereas only 8% and 7% received an adequate one respectively. 56% of the patients with PPF after THA and 57% of the patients with PPF after TKA reported about a previous fracture. Significant associations between the 10-year probability of a MOF and HF calculated by FRAX and PPF in THA and TKA were seen. The results of the present study show that FRAX might have the potential to estimate the PPF in patients following THA and TKA. FRAX should be calculated before and after THA or TKA in order to assess the risk and counsel patients. The data show a clear undertreatment of patients with PPF in respect to osteoporosis.

## Introduction

Postoperative periprosthetic fractures (PPF) are serious complications following total hip or knee arthroplasties (THA/TKA) and are associated with a dramatic health burden for the individual patients and high costs for health care systems. The incidence of PPF following primary THA or TKA reaches up to 5.5%^1–3^. It is expected that the incidence even rises further due to demographic changes in the developed world^4^. PPF following THA or TKA cause an increased morbidity and mortality and the majority of PPF need revision surgery^5^. Various risk factors for PPF are known among them are osteoporosis, female gender, older age, inflammatory arthritis, corticosteroid use and previous revision arthroplasty^6,7^. It has been shown that among patient who receive THA or TKA there is a high incidence of osteoporosis^8,9^. Furthermore, it has been shown that despite these numbers there is a lack of specific treatment of osteoporosis^10,11^. A fracture risk assessment (FRAX) tool, based on various epidemiological factors, has been developed to evaluate an individual fracture risk due to osteoporosis and guide diagnosis and medical treatment^12^. FRAX has been widely studied in patients with osteoporosis^13,14^. The aim of the present study is to assess the association between the 10-year probability of major osteoporotic fracture (MOF) and hip fracture (HF) calculated by FRAX and the probability of PPF following total hip and knee arthroplasty.

## Material and methods

Patients who were treated for PPF following THA or TKA between January 2015 and June 2019 in the AUVA Trauma Center Klagenfurt am Wörthersee were included in this study. Patients’ data was retrieved from a digital documentation system and demographic and medical data was extracted. Data collection was retrospectively. In case of missing information patients were interviewed by phone. Body mass index (BMI) was calculated using the formula weight/height^2^ (kg/m^2^).

Pre- and postoperative X-rays were seen by an arthroplasty fellowship trained orthopaedic surgeon (L.A.H). In case with missing pre- or postoperative X-rays patients were excluded. PPF of the acetabulum following THA were classified according to the Judet classification whereas in PPF of the femur following THA the Vancouver classification was applied^15–17^. PPF following TKA were classified either according to the Su classification or the classified according to Felix^18,19^. The date of the surgery was documented and an implant survival was calculated. Implant survival was defined as time of implantation to time of PPF (Table 1).

**Table 1:** Demographic data and risk factors associated with FRAX.

	Total	Male	Female
Number of patients (n)	167	38	129
Age at the time of fracture (years)	81.2 ± 9.9	74.9 ± 12.2	81.7 ± 9.8
Height in cm*	165.1 ± 6.6	178.4 ± 6.7	164. ± 5.3
Weight in kg*	67.3 ± 13.0	80.6 ± 12.5	66.1 ± 12.2
BMI in kg/m^2^*	24.6 ± 4.2	25.2 ± 6.7	24 ± 4.2
Previous fracture**	75 (44.91%)	12 (31.57%)	63 (48.83%)
Parent fractured hip**	1 (0.59%)	(0%)	1 (0.77%)
Current smoking**	6 (3.59%)	1 (2.63%)	5 (3.87%)
Glucocorticoids**	5 (2.99%)	0 (0%)	5 (3.87%)
Rheumatoid arthritis**	15 (8.98%)	4 (10.52%)	11 (8.52%)
Secondary osteoporosis**	36 (21.55%)	5 (13.15%)	31 (24.03%)
Alcohol 3 or more units/day**	0 (0%)	0 (0%)	0 (0%)
Implant survival in years	9.9 ± 6.6	8.4 ± 5.6	10.6 ± 7.1

*Mean + STD.

**Number of patients.

An individual 10-year probability of MOF and HF was calculated retrospectively using FRAX with the patients’ age at the date of initial surgery (in known cases) and at the time of PPF^20^. MOF is considered a fracture of the distal radius, proximal humerus or a vertebral body, whereas HF is a hip fracture. The following items are included in the FRAX questionnaire: age, sex, weight, height, previous fracture (a previous fracture denotes more accurately a previous fracture in adult life occurring spontaneously, or a fracture arising from trauma which, in a healthy individual, would not have resulted in a fracture), parent fractured hip (this enquires for a history of hip fracture in the patient's mother or father), current smoking, glucocorticoids, rheumatoid arthritis, secondary osteoporosis, alcohol 3 or more units/day and in case of a prior Dual-energy X-ray absorptiometry (DXA) scan the femoral neck bone mineral density (BMD) (g/cm^2^).

Based on the individual FRAX score a treatment suggestion was evaluated based on the National Osteoporosis Guideline Group (NOGG) guidelines such as pharmacological treatment in very high risk or high risk patients^21^. The NOGG guidelines are used as there are no specific guidelines available in Austria that would incorporate FRAX score in the treatment algorithms.

Statistical analyses were done using Microsoft® Excel for Mac (Version 16.16.27, Microsoft Corporation, Redmond, WA, USA). Bivariate correlation analysis was performed using the Pearson r correlation coefficient for PPF in patients with THA for implant survival and age, MOF, HF for female and male patients. Due to the small number of male patients (n = 3), the correlation coefficient for PPF with TKA these were just done for female patients. A *p* value of < 0.05 was considered significant.

The study protocol has been approved by the ethical committee of federal state of Carinthia (A 36/19). Informed consent was obtained from all subjects and/or their legal guardian(s). All methods were performed in accordance with the declaration of Helsinki.

## Results

167 patients (137 periprosthetic fractures in total hip arthroplasty and 30 periprosthetic fractures in total knee arthroplasty) were included in this study (see Table 1). The indication for primary THA was osteoarthritis in 112 (81%) %, hip fracture in 11 (8%), osteonecrosis of the hip in 9 (6.5%) and developmental dysplasia of the hip in 5 (3.6%) patients respectively. The indication for primary TKA was osteoarthritis in 30 patients (100%). Mean age of patients was 81.0 ± 10.7 years in PPF in THA and 76.5 ± 10.2 years in PPF in TKA respectively. Females represented 74% of the PPF in THA and 90% PPF in TKA. Mean height of patients was 168.0 ± 8.3 cm in PPF in THA and 165.0 ± 7.5 cm in PPF in TKA respectively. Mean weight of patients was 68.0 ± 13.2 kg in PPF in THA and 76.0 ± 14.1 kg in PPF in TKA respectively. Mean BMI of patients was 24.0 ± 3.6 kg/m^2^ in PPF in THA and 27.7 ± 4.7 kg/m^2^ in PPF in TKA respectively. In 106 patients (88 periprosthetic fractures in total hip arthroplasty and 18 periprosthetic fractures in total knee arthroplasty) the implant survival could be calculated. Results of the correlation analyses can be seen in Table 2 (Table 2).

**Table 2:** Correlation analysis between implant survival of THA and TKA, age, MOF and HF.

	Correlation of implant survival and patient’s age (at time of implantation)*	Correlation of implant survival and MOF*	Correlation of implant survival and HF*
PPF in THA and TKA (all patients)	− 0.50 (*p* < 0.01)	− 0.26 (*p* < 0.01)	− 0.27 (*p* < 0.01)
PPF in THA (female)	− 0.65 (*p* < 0.01)	− 0.38 (*p* < 0.01)	− 0.36 (*p* < 0.01)
PPF in THA (male)	− 0.36 (*p* < 0.05)	− 0.50 (*p* < 0.01)	− 0.50 (*p* < 0.05)
PPF in TKA (female)	− 0.31 (*p* < 0.05)	− 0.36 (*p* = 0.06)	− 0.35 (*p* = 0.07)

*Pearson r correlation coefficient.

### Periprosthetic fractures in THA

The most frequent fracture was a Vancouver type B2 periprosthetic fracture of the femur in 57%, followed by a Vancouver B1 periprosthetic fracture of the femur in 20% and Vancouver C periprosthetic fracture of the femur in 11%.

In 56% of the patients with periprosthetic fracture in THA there was a previous fracture in the patient’s history, whereas secondary osteoporosis was present in 26% and rheumatoid arthritis in 10%.

According to the NOGG guideline 88 of 137 (57%) were in need of treatment according to the FRAX values in THA patients. In the group of patients considered for treatment (very high risk or high risk) according to the NOGG guideline just 8% (7 out of 88) received adequate treatment pharmacologic for osteoporosis.

At the time of initial surgery the 10-year probability of a MOF was at a mean of 19.31% (± 14.5) and of a HF 9,8% (± 10.0).

At the time of postoperative periprosthetic fracture the 10-year probability of a MOF was at a mean of 26.8% (± 14.3) and of a HF 15.6% (± 10.7).

There were both a negative correlation of implant survival of THA and age in female patients (corr. coeff.—0.65, *p* < 0.01) and in male patients (corr. coeff.—0.36, *p* < 0.05). There was also a negative correlation of implant survival of THA and MOF probability in female patients (corr. coeff.—0.38, *p* < 0.01) and in male patients (corr. coeff.—0.5, *p* < 0.01). Furthermore, was a negative correlation of implant survival of THA and HF probability in female patients (corr. coeff.—0.36, *p* < 0.01) and in male patients (corr. coeff.—0.5, *p* < 0.01).

### Periprosthetic fractures in TKA

The most frequent fracture was a Su type II periprosthetic fracture of the distal femur in 83%, followed by Felix Type I fracture at the proximal tibia (7%) and a Su type II periprosthetic fracture in 4%. The majority of fractures occurred in female patients (27/30—90%). Therefore statistical analyses were just done in female patients. In 57% of the patients with periprosthetic fracture in TKA there was a previous fracture in the patient’s history. The second most frequent cause/risk factor was secondary osteoporosis (27%) and rheumatoid arthritis (10%). According to the NOGG guideline 43.3% (13/30) of the patients would require medical treatment (very high risk or high risk), whereas in fact only 1 (7%) received it before fracture.

At the time of initial surgery the 10-year probability of a MOF was at a mean of 18.0% (± 13.4) and of a HF 7.8% (± 8.9).

At the time of postoperative periprosthetic fracture the 10-year probability of a MOF was at a mean of 22.9% (± 13.1) and of a HF 11.3% (± 9.0).

In female patients there was a negative correlation of implant survival of TKA and age (corr. coeff.—0.31, *p* < 0.05), implant survival of TKA and MOF probability (corr. coeff.—0.36, *p* = 0.65) and implant survival of TKA and HF probability (corr. coeff.—0.35, *p* = 0.07).

### Periprosthetic fractures in TJA

In both male and female patients there was a negative correlation of implant survival of TJA and age (corr. coeff.—0.5, *p* < 0.01), implant survival of TJA and MOF probability (corr. coeff.—0.26, *p* < 0.01), and implant survival of TJA and HF probability (corr. coeff.—0.27, *p* < 0.01).

**Figure 1:**
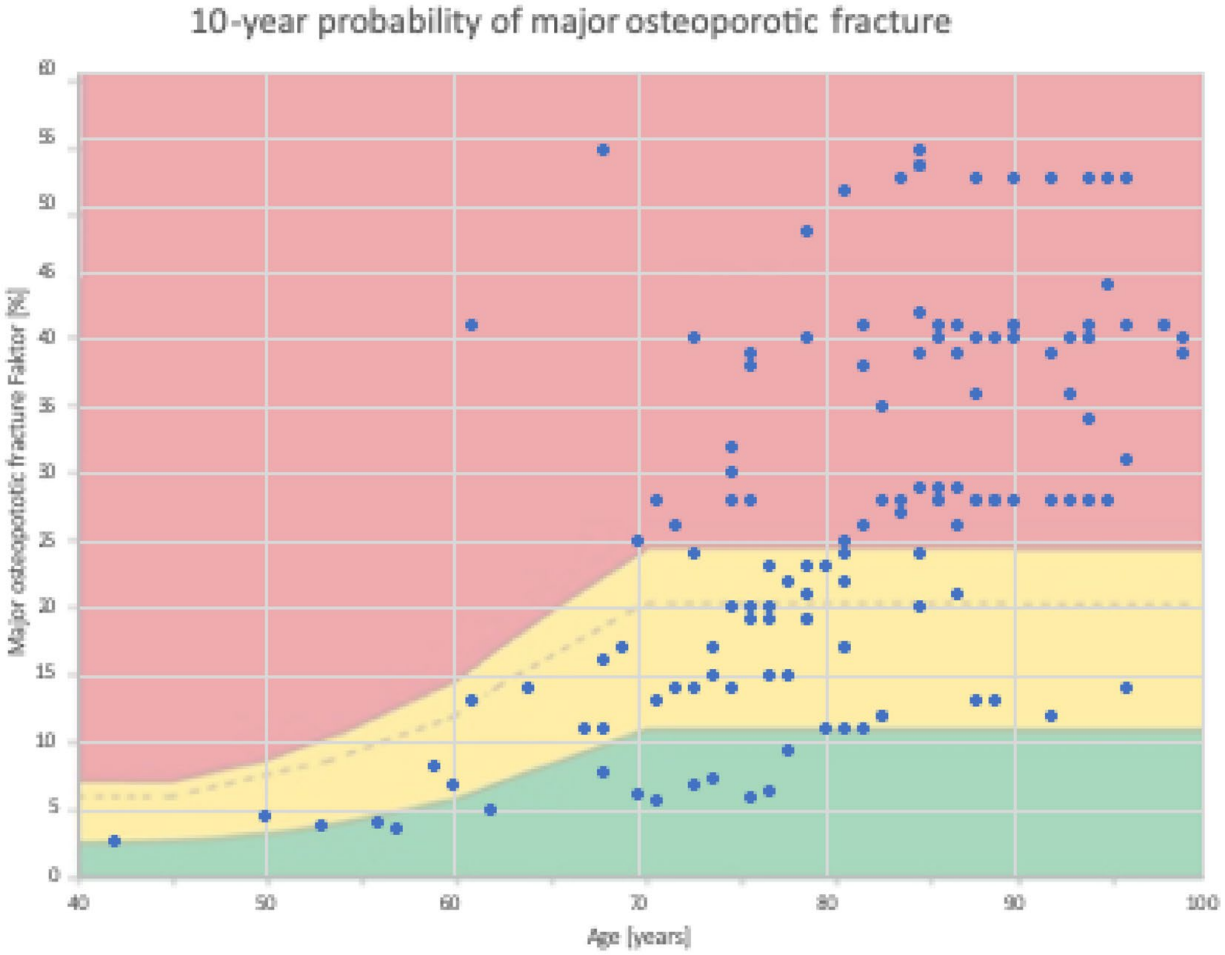
FRAX 10-year probability of MOF (major osteoporotic fracture). According to the NOGG the red area indicates that an osteoporosis treatment should be initiated, the yellow area indicates further diagnostic measures such as BMD and the green area lifestyle advice measures.

Results of the correlation analyses can be seen in Table 1 (Table 1), whereas Fig. 1 shows distribution of patients in respect to 10-year probability of MOF according to the NOGG guidelines (Fig. 1).

## Discussion

FRAX is a tool widely used to screen patients a risk of osteoporosis and osteoporotic fractures. In this study, FRAX has been evaluated among 167 patients with PPF following THA or TKA. Results show that the majority of patients who suffered PPF had an increased risk (10-year probability) of MOFs and HFs according to FRAX. In this respect in more than half of the patients, medical treatment would be indicated, which is in contrast to reality in which less then 10% received an adequate one.

This is in accordance with a recent study of the incidence of osteoporosis among TKA patients in which osteoporosis was present in 36% of the cases^8^. Furthermore, the same group invested osteoporosis specific medical treatment in TJA patients who had osteoporosis and found that just about 20% received adequate treatment^10^. Similar results were seen by Ha and Park who found an osteoporosis incidence of 50% among TKA patients of which only 15% received medical treatment for osteoporosis^11^. In a study of 268 elderly THA patients osteoporosis was present in 18% and osteopenia in 41%^9^. This points towards the need of additional osteoporosis screening not just at the time of implantation, but also on a regular base in the clinical follow-up to potentially prevent complications such as PPF. Furthermore, if indicated, medical treatment needs to be applied and patients counselled in this respect. In addition, patients should get a multidimensional treatment including fall prevention programs. It has been shown that lower scores of the 36-Item Short Form Survey (SF-36) subdomains of physical functioning and vitality are predictors for PPFs after primary TKA^22^.

The results of these study show significant associations between the 10-year probability of a MOF and HF calculated by FRAX and PPF in THA and TKA. Age and previous fracture, both components of the FRAX tool, seem to be major factors attributed to PPF risk which was seen in a high percentage among the studied patients and in previous publications^1,4,7,23^. Various studies showed that increased patient age is associated with the incidence of PPF^6,7^ Previous fractures are associated with an increased PPF risk^23^. As in osteoporosis where previous fractures are associated with subsequent fractures, a previous osteoporotic fracture seems to be an indicator of PPF^24^.

There are limitations to this study which includes the retrospective nature of data collection which causes missing data. As a such implant survival could not be calculated in all of the patients. Furthermore, important bone specific data is missing at all as no such data was available at the time of implantation. Furthermore, the study was limited to data that was retrieved from a documentation system, so additional information such as bone metabolic parameters, BMD or microCT are not available. The patient collective is quite heterogenous and includes to a majority THA patients, but only 30 TKA patients.

The results of this study can be seen as a preliminary report, however FRAX can easily be used in a clinical setting or even by patients themselves to estimate their current situation in respect to bone health. It was seen that the majority of patients that experienced PPFs are in need of osteoporosis specific treatment and have an increased likelihood of fractures. Further studies with an increased number of participants are needed to define the definitive value of FRAX to estimate PPF risk. At the moment however, it seems to be a good and easily accessible tool to be applied to define a status quo of bone health in patients before and following THA or TKA.

## Conclusions

The results of the present study show that FRAX might have the potential to be used as a tool to estimate individual PPF risk in patients following THA and TKA. The data shows that there is a association of the 10-year probability of MOF and HF and PPF. Furthermore, a clear undertreatment of patients with PPF in respect to osteoporosis has been seen. Further research is necessary to support the results of the present study.

## Data Availability

The datasets used and/or analyzed during the current study available from the corresponding author on reasonable request.
